# Molecular and transcriptional characterization of phosphatidyl ethanolamine-binding proteins in wild peanuts *Arachis duranensis* and *Arachis ipaensis*

**DOI:** 10.1186/s12870-019-2113-3

**Published:** 2019-11-09

**Authors:** Hanqi Jin, Xuemin Tang, Mengge Xing, Hong Zhu, Jiongming Sui, Chunmei Cai, Shuai Li

**Affiliations:** 1 College of Life Sciences, Key Lab of Plant Biotechnology in Universities of Shandong Province, Qingdao Agricultural University, Qingdao, 266109 China; 2 College of Agronomy, Qingdao Agricultural University, Qingdao, 266109 China

**Keywords:** Wild peanut, Phosphatidyl ethanolamine-binding protein (PEBP), Gene family, Flowering time, Plant architecture

## Abstract

**Background:**

Phosphatidyl ethanolamine-binding proteins (PEBPs) are involved in the regulation of plant architecture and flowering time. The functions of *PEBP* genes have been studied in many plant species. However, little is known about the characteristics and expression profiles of *PEBP* genes in wild peanut species, *Arachis duranensis* and *Arachis ipaensis****,*** the diploid ancestors of cultivated peanuts.

**Results:**

In this study, genome-wide identification methods were used to identify and characterize a total of 32 peanut *PEBP* genes, 16 from each of the two wild peanut species, *A. duranensis* and *A. ipaensis*. These *PEBP* genes were classified into 3 groups (*TERMINAL FLOWER1*-like, *FLOWERING LOCUS T*-like, and *MOTHER OF FT AND TFL1*-like) based on their phylogenetic relationships. The gene structures, motifs, and chromosomal locations for each of these *PEBPs* were analyzed. In addition, 4 interchromosomal duplications and 1 tandem duplication were identified in *A. duranensis*, and 2 interchromosomal paralogs and 1 tandem paralog were identified in *A. ipaensis*. Ninety-five different *cis*-acting elements were identified in the *PEBP* gene promoter regions and most genes had different numbers and types of *cis*-elements. As a result, the transcription patterns of these *PEBP* genes varied in different tissues and under long day and short day conditions during different growth phases, indicating the functional diversities of *PEBPs* in different tissues and their potential functions in plant photoperiod dependent developmental pathways. Moreover, our analysis revealed that *AraduF950M*/*AraduWY2NX* in *A. duranensis*, and *Araip344D4*/*Araip4V81G* in *A. ipaensis* are good candidates for regulating plant architecture, and that *Aradu80YRY, AraduYY72S,* and *AraduEHZ9Y* in *A. duranensis* and *AraipVEP8T* in *A. ipaensis* may be key factors regulating flowering time.

**Conclusion:**

Sixteen *PEBP* genes were identified and characterized from each of the two diploid wild peanut genomes, *A. duranensis* and *A. ipaensis*. Genetic characterization and spatio-temporal expression analysis support their importance in plant growth and development. These findings further our understanding of *PEBP* gene functions in plant species.

## Background

Flowering time is a critical factor influencing the production of offspring in plants. Thus, plants have evolved complex systems regulating the transition from the vegetative to the reproductive phase. The precise adjustment of flowering time is controlled by both internal and environmental cues [1–4]. Several pathways regulating flowering time have been identified, such as photoperiod, vernalization, giberellic acid, and autonomous pathways [1, 2, 5–7]. Moreover, numerous molecular regulatory components have been shown to participate in these flowering regulatory pathways. For example, many MADS-box family members and phosphatidyl ethanolamine-binding proteins (PEBPs) have been shown to participate in the switch from the shoot apical meristem to the inflorescence meristem [1, 8, 9].

The *PEBP* gene family is an ancient, conserved set of genes encoding proteins that are highly similar in all eukaryote kingdoms, including bacteria, animals, and plants [10–12]. PEBPs were identified by their preference for binding phosphatidyl ethanolamine lipids over other phospholipids [13]. Previous investigation into the functions of *PEBP* genes revealed that *PEBPs* encode proteins that are involved in multiple signal pathways regulating growth and differentiation in many species [12, 14, 15]. In plants, *PEBP* genes mainly participate in flowering time and plant architecture regulation [11, 16, 17]. Many recent studies have investigated individual *PEBP* genes, however, the complete *PEBP* family has been studied in only a few plant species, such as Arabidopsis, soybean, rice, maize, and cotton [8, 18–20].

In plants, *PEBP* family genes are generally classified into three groups: *TERMINAL FLOWER1* (*TFL1*)-like, *FLOWERING LOCUS T* (*FT*)-like, and *MOTHER OF FT AND TFL1* (*MFT*)-like sub-families [1, 8, 18, 21, 22]. In Arabidopsis, the *FT*-like sub-family contains two members, *FT* and *TWIN SISTER OF FT* (*TSF*), both of which promote the transition from vegetative to reproductive growth [1, 23, 24]. *FT* is a circadian clock gene, and its protein has been shown to move from the leaves to the shoot apical meristem and interact with the transcription factor FD to accelerate flowering [25–29]. *TSF*, the closest homolog of *FT*, has similar functions. Overexpression of *TSF* promotes flowering and *tsf* mutants have delayed flowering time phenotypes [1, 30]. The *TFL1*-like sub-family has three members, *TFL1*, *Arabidopsis thaliana CENTRORADIALIS* (*ATC*), and *BROTHER OF FT* (*BFT*), all of which have been reported to delay flowering time. *TFL1* is involved in the regulation of flowering time and plant architecture. Mutation of *TFL1* causes early flowering time and determinate growth habit [1, 31]. *ATC* is a short-day induced floral inhibitor, and its protein travels through the vasculature to the shoot apex to influence flowering time [1, 32]. *BFT* shows a diurnally oscillating expression pattern that peaks in the early evening, similar to *FT*. Overexpression of *BFT* causes delayed flowering time and severe floral defects, while knock-down of *BFT* has no effect on flowering time. Thus, *BFT* works redundantly in the determination of flowering time [1, 33]. The *MFT*-like sub-group contains only one member, *MFT*, which has weak *FT*-like activity and mainly participates in the seed germination signal pathway [34, 35].

Peanut is an important oil legume throughout the world. The cultivated peanut is an allotetraploid (AABB, 2*n* = 4*x* = 40) and is thought to have been derived from hybridization and polyploidization of two diploid species, *A. duranensis* (AA genome) and *A. ipaensis* (BB genome) [36–39]. Improving plant architecture and flowering time could help to increase peanut production. Analysis of functional genes can help identify modifications that can be made to peanut cultivars in order to increase yields. The investigation of wild peanut genes will provide essential information for further functional characterization of cultivated peanut genes [40, 41]. In this study, *PEBP* genes were identified and characterized from two wild peanut species, *A. duranensis* and *A. ipaensis*. Many characteristics of these *PEBP* genes were analyzed, including gene evolutionary relationships, gene structures, conserved motifs, and gene expression patterns. Our findings will enable further characterization of *PEBP* gene and protein functions in peanuts.

## Methods

### Plant materials and growth conditions

Wild *A. duranensis* PI219823 and *A. ipaensis* PI468322 species were used for gene expression analysis. Peanut seeds were germinated in tap water and then planted in pots in growth chambers with different photoperiods. The growth conditions were set as 16 h 24 °C light/8 h 24 °C dark and 10 h 24 °C light/14 h 24 °C dark cycles for long day and short day photoperiods, respectively. The humidity was controlled at approximately 30%. Plant leaves were sampled 2 h after lights-on at different growth stages. Stage 1 to stage 6 (S1-S6) were considered to be when the first two, the third, the fourth, the fifth, the sixth, and the seventh leaves were fully expanded, respectively. To confirm the expression levels of *PEBP* genes in different tissues, the cultivated peanut Tiffrunner and wild peanuts were grown in the field in Qingdao, China, and different tissues were collected for analysis. The samples were stored at − 80 °C before RNA extraction.

### Identification of peanut *PEBP* members

The amino acid sequence of the PEBP conserved domain (PF01161) and PEBP amino acid sequences from Arabidopsis [1] and soybean [8] were used as blast queries against the peanut genome database (https://www.peanutbase.org/). All output genes were analyzed using the Pfam database (http://pfam.xfam.org/search) and the National Center for Biotechnology Information (NCBI) in order to confirm the conserved PEBP domains. Genes without conserved domain sequences were discarded. The protein molecular weight and theoretical iso-electric points were determined using ProtParam (https://web.expasy.org/protparam/). The subcellular localizations of PEBPs were predicted using the ProtComp tool (http://linux1.softberry.com/berry.phtml?topic=protcomppl&group=programs&subgroup=proloc).

### Phylogenetic relationship analysis

The full-length PEBP amino acid sequences from two wild peanut species, cultivated peanut, Arabidopsis, soybean, common bean, and medicago [8] were aligned using Clustal-X2. The alignment results were used to construct a phylogenetic tree using the Neighbor-Joining method in MEGA 7 [42, 43].

### Analyses of *PEBP* gene structures and conserved motifs

The exon-intron organizations of wild peanut *PEBPs* were determined with the Gene Structure Display Server program (GSDS) using the coding domain sequences (CDS) and genomic sequences obtained from the peanut genome database [44]. The peanut PEBP conserved motifs were analyzed using MEME tools (http://meme-suite.org/) with the following parameters: a maximum number of 15 motifs and an optimum motif width of 6–50 amino acid residues.

### Analyses of chromosomal localization, gene duplication, synteny, and *cis*-acting elements

To analyze chromosomal distribution, *PEBP* gene positions were obtained from the peanut genome database and mapped to the physical chromosome positions. Synteny analysis between the soybean, the common bean, and the peanut was carried out as described by Zhang et al. [45]. To analyze gene duplication, peanut PEBP sequences were clustered using OrthoMCL software and homologous relationships were determined using the Circos software [46, 47]. The *cis*-acting elements of peanut *PEBP* genes were predicted by PlantCARE (http://bioinformatics.psb.ugent.be/webtools/plantcare/html/) [48], using the promoter regions 2 kb upstream of the translation initiation codons of each *PEBP* gene.

### Subcellular localization analysis

The subcellular localizations of representative wild peanut *PEBP* genes were analyzed as described by Li et al. [49]. Each full-length *PEBP* gene and GFP were amplified and cloned into modified pCAMBIA1300 vectors. The constructs were then transiently expressed in *Nicotiana benthamiana* leaves using the Agrobacterium-mediated infiltration method.

### RNA extraction and expression analysis

RNA extraction and quantitative real-time PCR (qRT-PCR) were performed as described by Li et al. [49]. Briefly, RNeasy mini kits (Qiagen) were used to isolate total plant RNA. First-strand cDNAs synthesis was performed using SuperScript II reverse transcriptase (Promega) and 1.5 μg total RNA from each sample. qRT-PCR was performed with a LightCycler480 machine (Roche Diagnostics) using LightCycler 480 SYBR Green I Master Kit (Roche Diagnostics). The qRT-PCR amplification program was as follows: 94 °C for 10 s, 58 °C for 10 s, and 72 °C for 10 s, for 40 cycles. The gene expression levels were normalized to the wild peanut *Actin*-expressing gene (*AraduW2Y55* or *AraipFY50U*). For gene expression analysis in the cultivated peanut, gene expression levels were normalized to the *Actin*-expressing gene, as described by Sui et al. [50]. Each sample was analyzed using three biological replicates. All the primers used in this study are listed in Additional file 10. To determine the transcription patterns of *PEBP* genes in various tissues, the RNA-seq datasets of 22 different tissues from cultivated peanut were obtained from the peanut database (https://peanutbase.org/gene_expression). The 22 tissues were identified as described by Clevenger et al. [51] and are as follows: ‘Seedling Leaves’ (seedling leaves 10 days after emergence), ‘Main Stem Leaves’, ‘Lateral Stem Leaves’, ‘Vegetative Shoot Tip’ (from the main stem), ‘Reproductive Shoot Tip’ (from the first lateral leaf), ‘Roots’ (10 day-old roots), ‘Nodule Roots’ (25 day-old nodules), ‘Flowers’ (perianth), ‘Pistils’ (gynoecium), ‘Stamens’ (androecium), ‘Aerial Gyn Tip’ (aerial gynophore tip), ‘Sub Gyn Tip’ (subterranean gynophore tip), ‘PodPt1’ (pattee stage 1 pod), ‘StalkPt1’ (pattee stage 1 stalk), ‘PodPt3’ (pattee stage 3 pod), ‘Pericarp Pattee5’ (pattee stage 5 pericarp), ‘Seed Pattee5’ (pattee stage 5 seed), ‘Pericarp Pattee6’ (pattee stage 6 pericarp), ‘Seed Pattee6’ (pattee stage 6 seed), ‘Seed Pattee7’ (pattee stage 7 seed), ‘Seed Pattee8’ (pattee stage 8 seed), and ‘Seed Pattee10’ (pattee stage 10 seed). The *A. hypogaea* gene expression profiles were mapped to *A. duranensis* and *A. ipaensis* for heat map analysis [51–53].

## Results

### Identification of *PEBP* genes in two wild peanut species

To identify *PEBP* genes expressed in the wild peanut species *A. duranensis* and *A. ipaensis*, the amino acid sequences of the PEBP conserved domain (PF01161) and the full-length PEBP protein sequences from Arabidopsis and soybean were used as blast queries against the peanut genome database. Pfam and NCBI tools were then used to confirm the conserved PEBP domains in these candidate *PEBP* genes. In total, 32 *PEBP* genes were identified from the two wild peanut genomes (Table 1). Multiple *PEBP* gene characteristics were analyzed using the genomic, CDS, and amino acid sequences (Table 1). The genomic lengths of wild peanut *PEBP* genes ranged from 989 bp (*AraduP7QP3*) to 8770 bp (*AraduWY2NX*), CDS lengths ranged from 327 bp (*AraduWY2NX*) to 630 bp (*AraduG0NJW*), and the deduced number of amino acids ranged from 108 to 209. The molecular weights and isoelectric points for each PEBP were predicted (AraduG0NJW was discarded due to lack of related information). The molecular weights ranged from 12,058.74 to 20,482.26 kDa and the isoelectric points ranged from 4.93 to 9.68. In addition, the PEBP sub-cellular localizations were predicted. No information was found about the localization of Aradu23179, Aradu60NUI, or AraipV0B0S*.* All other wild peanut PEBPs were predicted to be localized in both the cytoplasm and the nucleus (Table 1), similar to soybean PEBPs [8]. The subcellular localizations of several PEBPs in *A. duranensis*, including AraduWY2NX, AraduYY72S, and AraduQIZ46, and in *A. ipaensis,* including Araip4V81G, AraipVEP8T and AraipA8S33 were identified. The results confirmed their subcellular localizations in cytoplasm and nucleus (Additional file 1).

**Table 1:** *PEBP* members identified from two wild peanut species

Gene ID	Chr	Genomic Length (bp)	CDS Length(bp)	No. of AA	Mol.Wt (kDa)	pI	Subcellular Localization	Gene Family
*AraduEHZ9Y*	A02	5124	507	168	18,645.2	6.9	Cytoplasm and Nucleus	*FT*-like
*Aradu23179*	A02	1027	510	169	18,629.02	5.37	N/A	*MFT-*like
*Aradu60NUI*	A02	1439	528	175	19,629.32	4.93	N/A	*MFT-*like
*AraduF950M*	A02	1985	444	147	16,315.35	6.57	Cytoplasm and Nucleus	*TFL1-*like
*AraduZ8JSI*	A03	4282	531	176	19,253.3	8.57	Cytoplasm and Nucleus	*MFT-*like
*AraduA9H9T*	A04	1968	543	180	20,293.04	6.51	Cytoplasm and Nucleus	*FT-*like
*AraduA4ISL*	A04	2826	543	180	20,473.5	7.86	Cytoplasm and Nucleus	*FT-*like
*Aradu1I7E9*	A04	1175	534	177	19,831.56	6.73	Cytoplasm and Nucleus	*FT-*like
*AraduP7QP3*	A05	989	486	161	18,175.77	9.15	Cytoplasm and Nucleus	*TFL1-*like
*AraduWY2NX*	A06	8770	327	108	12,058.74	5.14	Cytoplasm and Nucleus	*TFL1-*like
*Aradu80YRY*	A06	1984	537	178	19,874.48	8.54	Cytoplasm and Nucleus	*FT-*like
*AraduA6WCN*	A08	3143	489	162	18,224.68	9.12	Cytoplasm and Nucleus	*FT-*like
*AraduRJP5K*	A08	1296	495	164	18,620.2	9.21	Cytoplasm and Nucleus	*TFL1-*like
*AraduQIZ46*	A10	2030	537	178	19,345.85	7.8	Cytoplasm and Nucleus	*MFT-*like
*AraduG0NJW*	A10	3071	630	209	N/A	N/A	Cytoplasm and Nucleus	*FT-*like
*AraduYY72S*	A10	1652	519	172	19,688.48	8.9	Cytoplasm and Nucleus	*FT-*like
*AraipV0B0S*	B02	1134	510	169	18,692.12	5.37	N/A	*MFT-*like
*Araip344D4*	B02	1513	432	143	16,183.4	9.62	Cytoplasm and Nucleus	*TFL1-*like
*AraipWF9GZ*	B03	4518	531	176	19,248.28	8.93	Cytoplasm and Nucleus	*MFT-*like
*AraipPC28F*	B04	1810	543	180	20,391.15	6.58	Cytoplasm and Nucleus	*FT-*like
*AraipU9HL1*	B04	2806	525	174	19,750.6	6.9	Cytoplasm and Nucleus	*FT-*like
*AraipT1SIZ*	B04	1298	534	177	19,845.59	6.73	Cytoplasm and Nucleus	*FT-*like
*AraipYA5YU*	B05	6384	531	176	19,780.57	6.82	Cytoplasm and Nucleus	*FT-*like
*AraipA5PDN*	B05	995	486	161	18,153.78	9.15	Cytoplasm and Nucleus	*TFL1-*like
*Araip4V81G*	B06	1414	417	138	15,444.45	6.05	Cytoplasm and Nucleus	*TFL1-*like
*AraipZJ9GZ*	B06	4212	546	181	20,482.26	9.04	Cytoplasm and Nucleus	*FT-*like
*AraipV23ZE*	B06	1446	441	146	16,362.59	9.68	Cytoplasm and Nucleus	*FT-*like
*Araip03WUR*	B07	2576	498	165	18,585.18	6.82	Cytoplasm and Nucleus	*FT-*like
*AraipT6XJY*	B08	1387	543	180	20,385.32	9.24	Cytoplasm and Nucleus	*TFL1-*like
*AraipWWI38*	B09	2632	522	173	19,214.79	7.82	Cytoplasm and Nucleus	*FT-*like
*AraipA8S33*	B10	1766	537	178	19,495.09	8.53	Cytoplasm and Nucleus	*MFT-*like
*AraipVEP8T*	B10	1535	522	173	19,275.74	6.83	Cytoplasm and Nucleus	*FT-*like

*Chr* chromosome number, *AA* amino acid, *Mol.Wt* molecular weight, *pI* isoelectric point, *N/A* not applicable

### Chromosomal localization analysis

**Fig. 1:**
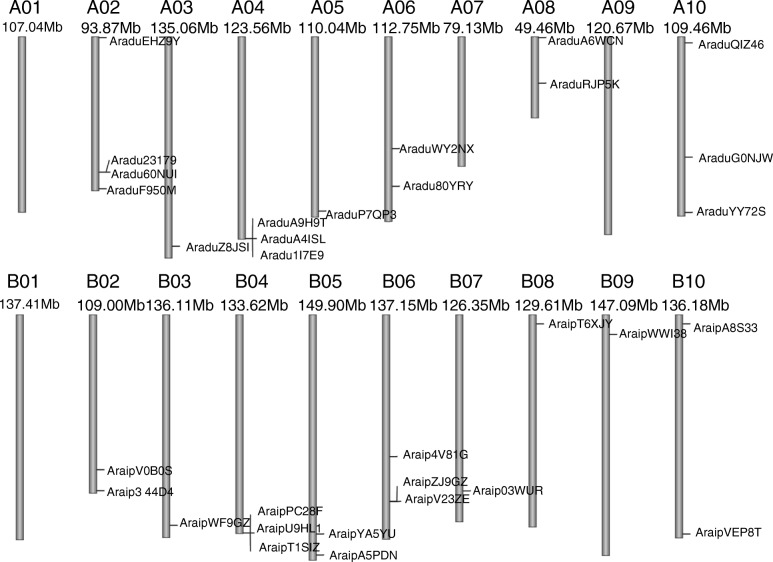
Chromosomal distribution of *PEBP* genes in *A. duranensis* (AA genome) and *A. ipaensis* (BB genome)

To determine the chromosomal locations of wild peanut *PEBPs*, the *PEBP* genes were mapped to the related chromosome positions using the peanut genome database (Fig. 1). The chromosome distribution map revealed the positions of the 16 *PEBP* genes from each of the two wild peanut species (Fig. 1 and Table 1). For the AA genome wild specie, *PEBP* genes were distributed on 7 of the 10 chromosomes and no *PEBP* genes were found on chromosome 1, 7, or 9. Chromosome 2 contained the most *PEBP* genes, 4 in total (Fig. 1). In contrast, 9 of the 10 chromosomes in the BB wild peanut genome contained *PEBP* genes, all except for chromosome 1. Chromosomes 4 and 6 contained the most *PEBP* genes in the BB genome, with 3 *PEBP* genes on each (Fig. 1). Among these *PEBP* genes, most were located in the chromosome arms (Fig. 1). Only four genes, including *AraduWY2NX, AraduRJP5K, AraduG0NJW,* and *Araip4V81G*, were found close to the middle of the chromosome (Fig. 1).

### Classification and phylogenetic analysis of *PEBP* genes

**Fig. 2:**
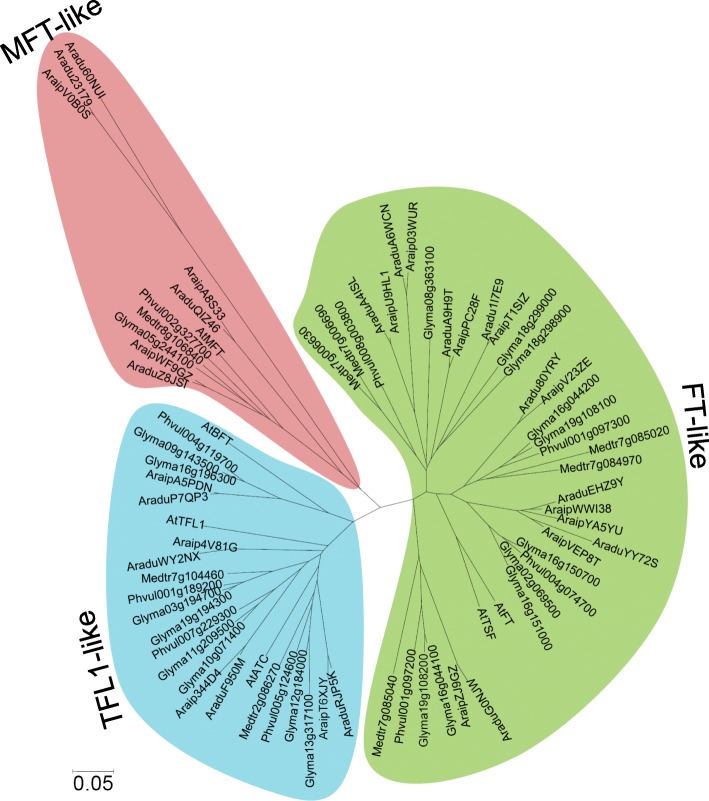
Evolutionary relationship analysis of PEBP proteins from two wild peanut species. PEBP amino acid sequences from wild peanut species, Arabidopsis, soybean, common bean, and medicago were used to construct a phylogenetic tree

In plants, *PEBP* genes can be grouped into 3 sub-families, *TFL1*-like, *FT*-like, and *MFT*-like, according to their gene structures [1, 8]. To classify the wild peanut *PEBP* genes into different sub-families and infer information based on the well-studied homologous *PEBP* genes from other plant species, a phylogenetic tree was constructed using *PEBP* genes from wild peanuts, Arabidopsis, soybean, common bean, and medicago [8]. The phylogenetic relationship analysis classified the wild peanut *PEBP* genes into 3 sub-families (Fig. 2 and Table 1). Among these members, 8 *PEBP* genes belonged to the *TFL1*-like sub-family, 17 members were grouped into the *FT*-like sub-family, and 7 *PEBPs* were classified into the *MFT*-like sub-family. The AA genome contained 8 *FT*-like, 4 *TFL1*-like, and 4 *MFT*-like genes. In contrast, the BB genome had 9 *FT*-like, 4 *TFL1*-like, and 3 *MFT*-like members, likely reflecting the functional differentiation of genes in the AA and BB genomes. Because the cultivated peanut is an allotetraploid derived from hybridization and polyploidization of *A. duranensis* and *A. ipaensis*, cultivated peanut (*A. hypogaea*) *PEBP* genes were also investigated and 31 *PEBP* members were identified (Additional file 2). Phylogenetic analysis revealed that the *A. hypogaea* genome contained 8 *TFL1*-like, 16 *FT*-like, and 7 *MFT*-like members (Additional file 2).

**Fig. 3:**
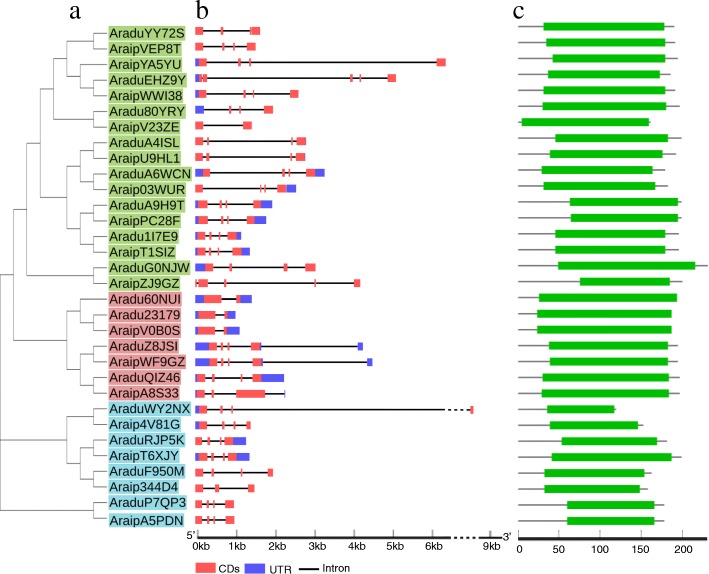
Phylogenetic and structure analysis of *PEBP* genes from two wild peanut species. **a** Phylogenetic relationship analysis of wild peanut PEBP proteins. The green, pink, and light blue colors indicate *TFL1*-like, *MFT*-like, and *FT*-like sub-families, respectively. **b** The exon-intron organizations of *PEBP* genes. UTRs, exons, and introns are represented by dark blue, red, and black lines, respectively. **c** The green boxes indicate conserved PEBP domains in each of the different peanut PEBP proteins

Many orthologous gene pairs have been identified between the AA and BB genomes in wild peanuts [40, 41, 54, 55]. Thus, orthologous *PEBP* gene pairs were investigated using a phylogenetic tree generated with wild peanut PEBP amino acid sequences (Fig. 3a). Fifteen orthologous *PEBP* gene pairs (all except for *AraipYA5YU* and *Aradu60NUI*) were classified into the same cluster (Fig. 3a). Most of the gene pairs have highly similar CDS and protein sequences (Fig. 3a, and Table 2), suggesting that orthologous genes have similar functions. Chromosomal localization analysis showed that 11 of the 15 orthologous gene pairs were found on the syntenic locus of *A. duranensis* and *A. ipaensis* chromosomes (Fig. 1 and Table 2). However, *AraduEHZ9Y*, *AraduA6WCN,* and *AraduG0NJW* were found to be located on different chromosomes than their related orthologous genes in *A. ipaensis*. *AraduRJP5K* was located in the middle part of chromosome 8, while its orthologous gene, *AraipT6XJY,* was located in the chromosome arm (Fig. 1 and Table 2). This suggests that chromosomal rearrangement might have occurred in the diploid peanut genomes [54, 56].

**Table 2:** *AdPEBP* and *AiPEBP* orthologous genes

Gene pairs	Groups	Chr	CDS	Protein
			Identity (%)	Identity (%)
*AraduYY72S-AraipVEP8T*	*FT*-like—*FT*-like	A10-B10	93.06%	89.60%
*AraduEHZ9Y-AraipWWI38*	*FT*-like—*FT*-like	A02-B09	93.64%	91.91%
*Aradu80YRY-AraipV23ZE*	*FT*-like—*FT*-like	A06-B06	97.41%	77.53%
*AraduA4ISL-AraipU9HL1*	*FT*-like—*FT*-like	A04-B04	97.90%	97.70%
*AraduA6WCN-Araip03WUR*	*FT*-like—*FT*-like	A08-B07	93.78%	93.33%
*AraduA9H9T-AraipPC28F*	*FT*-like—*FT*-like	A04-B04	97.97%	97.22%
*Aradu1I7E9-AraipT1SIZ*	*FT*-like—*FT*-like	A04-B04	98.31%	98.87%
*AraduG0NJW-AraipZJ9GZ*	*FT*-like—*FT*-like	A10-B06	98.57%	74.16%
*Aradu23179-AraipV0B0S*	*MFT*-like—*MFT*-like	A02-B02	97.65%	98.22%
*AraduZ8JSI-AraipWF9GZ*	*MFT*-like—*MFT*-like	A03-B03	96.80%	96.59%
*AraduQIZ46-AraipA8S33*	*MFT*-like—*MFT*-like	A10-B10	96.65%	94.94%
*AraduP7QP3-AraipA5PDN*	*TFL1*-like—*TFL1*-like	A05-B05	98.35%	98.14%
*AraduWY2NX-Araip4V81G*	*TFL1*-like—*TFL1*-like	A06-B06	99.35%	96.30%
*AraduRJP5K-AraipT6XJY*	*TFL1*-like—*TFL1*-like	A08-B08	96.62%	88.20%
*AraduF950M-Araip344D4*	*TFL1*-like—*TFL1*-like	A02-B02	92.79%	91.16%

*Chr* chromosome number

### Exon-intron structures and conserved domains of *PEBP* genes

**Fig. 4:**
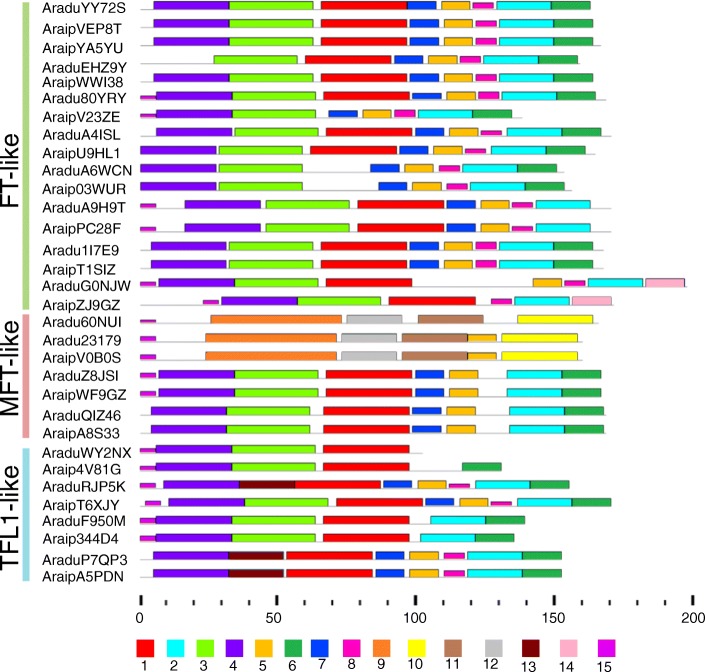
Wild peanut PEBP protein motifs identified by MEME. The colored boxes, numbered 1–15, indicate different motifs

Classical *PEBP* members have a conserved 4-exon gene structure [19]. To determine the gene structures of wild peanut *PEBP* genes, the Gene Structure Display Server program was used to investigate *PEBP* exon-intron organizations [44]. Our findings revealed that 23 of the 32 *PEBP* members had the 4-exon conserved gene structure, while 9 genes displayed inconsistent organization (Fig. 3b). For these 9 genes, the *FT*-like members *AraduEHZ9Y*, *Aradu80YRY*, *AraipV23ZE*, and *AraipZJ9GZ* contained 5, 3, 2, and 5 exons, respectively. The *MFT*-like genes *Aradu60NUI*, *Aradu23197*, *Araduv0B0S,* and *AraipA8S33* had 2, 2, 2, and 3 exons, respectively, and the *TFL1*-like gene *Araip344D4* had 3 exons (Fig. 3b). In addition, the PEBP domains in each of the wild peanut PEBPs were analyzed and the lengths of the PEBP domains were found to be more than half of each PEBP protein, except for AraipZJ9GZ (Fig. 3c and Additional file 3). To further analyze PEBP structures, the conserved motifs of PEBP proteins were investigated. Fifteen distinct motifs were found in the 32 wild peanut PEBPs (Fig. 4 and Additional file 4). However, no single motif was found on all PEBP proteins, indicating their function diversity.

### Analysis of *PEBP* gene duplication and synteny

**Fig. 5:**
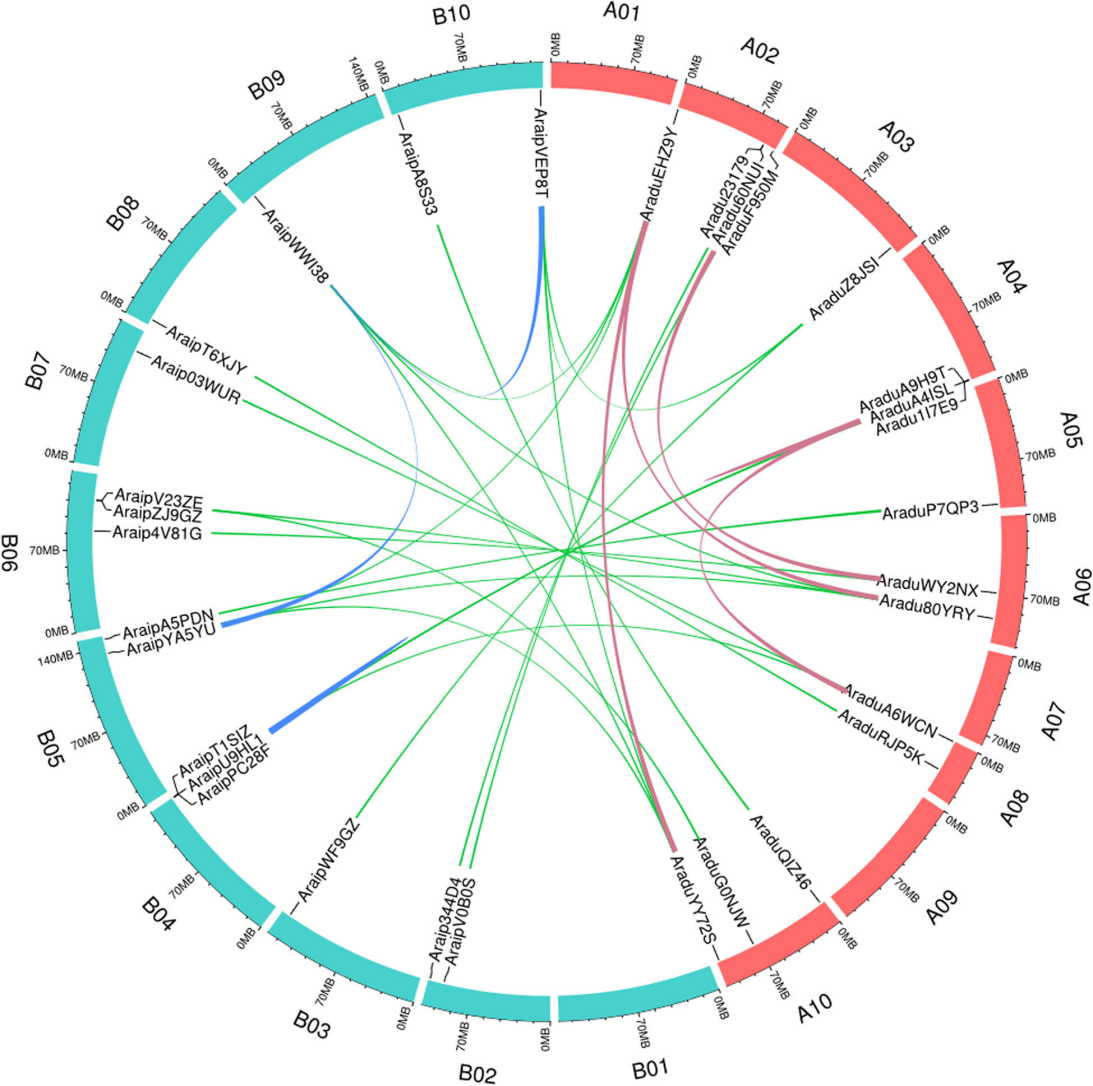
Homology analysis of *A. duranensis* and *A. ipaensis PEBP* genes*.* Green lines indicate homologous gene pairs between *A. duranensis* and *A. ipaensis*. Red lines indicate duplicated gene pairs in *A. duranensis* and blue lines indicate duplication events in *A. ipaensis*

Polyploidy is common in flowering plants, and gene duplication often occurs during gene evolution [57], thus a homology analysis of the wild peanut genomes was performed. Many paralogous genes were found in both *A. duranensis* and *A. ipaensis* (Fig. 5). Four interchromosomal duplications and one tandem duplication were identified in *A. duranensis*. Three interchromosomal duplications, *AraduYY72S/AraduEHZ9Y*, *Aradu80YRY/AraduEHZ9Y*, and *AraduA6WCN/AraduA4ISL*, belonged to the *FT*-like sub-family and *AraduWY2NX/AraduF950M* belonged to the *TFL1*-subfamily. The tandem duplication was composed of three genes: *AraduA9H9T*, *AraduA4ISL,* and *Aradu1I7E9*. Two interchromosomal paralogs, *AraipWWI38/AraipVEP8T* and *AraipWWI38/AraipYA5YU*, and one tandem duplication were found in *A. ipaensis*. The two duplicated gene pairs belonged to the *FT*-like sub-class, and the tandem paralog contained three members: *AraipPC28F*, *AraipT1SIZ,* and *AraipU9HL1*. In addition, a homology analysis was also perfomed between *A. duranensis* and *A. ipaensis* and 25 homologous gene pairs were identified. Among these gene pairs, the tandem duplicates *AraduA9H9T*, *AraduA4ISL,* and *Aradu1I7E9* in *A. duranensis* were found to be homologous with the tandem paralogs *AraipPC28F*, *AraipT1SIZ,* and *AraipU9HL1* in *A. ipaensis* (Fig. 5).

Arabidopsis *TFL1* regulates plant architecture and its homologous genes in legumes are syntelogs with conserved functions [1, 58–61]. To investigate the potential functions of wild peanut *TFL1*-like genes, the genomic regions surrounding soybean growth habit gene *GmDt1 (Glyma19g194300)*, common bean growth habit gene *PvTFL1y (Phvul001g189200)*, and wild peanut *TFL1* homologs were compared to determine whether they were syntelogs. Syntenys between wild peanut genes *AraduF950M*, *AraduWY2NX*, *Araip344D4*, *Araip4V81G,* soybean *GmDt1*, and common bean *PvTFL1y* were found in all regions compared (Additional file 5), indicating that these genes may have evolved from the same origin and that these wild peanut genes might participate in plant architecture regulation.

### Analysis of *cis*-acting elements in wild peanut *PEBP* gene promoter regions

To investigate the regulation of wild peanut *PEBP* gene expression, *cis*-acting elements in the promoter regions 2 kb upstream of the translation initiation codons were analyzed. In total, 95 types of *cis*-acting elements were found upstream of these *PEBP* genes, of which 56 were predicted to have putative functions, including 7 development related elements, 4 environmental stress related elements, 10 hormone-responsive elements, 26 light-responsive elements, 5 promoter related elements, and 4 site-binding related elements (Table 3 and Additional file 6). Among these functional *cis*-acting elements, the light-responsive elements were the most abundant type in each *PEBP* promoter, and all of the *PEBP* genes contained hormone-responsive elements, light-responsive elements, and promoter related elements, suggesting that all of the *PEBPs* are involved in related signaling pathways (Table 3). Moreover, the promoter-related element TATA-box was found in all 32 wild peanut *PEBP* promoter regions, suggesting that the TATA-box is necessary for expression of *PEBP* genes. Thirty-one *PEBP* promoters contained light-responsive element Box 4, except for the *FT*-like gene *AraipYA5YU* (Additional file 6). In addition, the types and numbers of *cis*-acting elements showed diverse distribution among the *PEBP* promoters (Table 3 and Additional file 6), suggesting that *PEBP* genes are functionally diverse and might work in different signaling pathways.

**Table 3:** *Cis*-acting elements in the promoter region of each wild peanut *PEBP* gene

Gene name	Gene Family	Development related elements	Environmental stress related elements	Hormone-responsive elements	Light-responsive elements	Promoter related elements	Site-binding related elements	Others
*AraduEHZ9Y*	*FT*-like	0	1	5	10	2	0	16
*AraduF950M*	*TFL1*-like	1	0	5	9	2	0	12
*AraduZ8JSI*	*MFT*-like	3	2	3	8	2	1	18
*AraduA9H9T*	*FT*-like	2	2	3	9	2	0	12
*AraduA4ISL*	*FT*-like	0	1	4	6	3	0	18
*Aradu1I7E9*	*FT*-like	0	3	5	6	2	1	17
*AraduP7QP3*	*TFL1*-like	1	2	1	7	2	1	13
*AraduWY2NX*	*TFL1*-like	2	2	5	7	2	0	12
*Aradu80YRY*	*FT*-like	2	2	5	9	2	0	13
*AraduA6WCN*	*FT*-like	2	1	1	5	3	1	15
*AraduRJP5K*	*TFL1*-like	0	1	5	8	2	2	18
*AraduQIZ46*	*MFT*-like	2	1	5	7	2	2	17
*AraduG0NJW*	*FT*-like	1	1	3	7	2	0	13
*Aradu60NUI*	*MFT*-like	1	2	4	9	2	1	15
*Aradu23179*	*MFT*-like	2	1	5	7	2	1	15
*AraduYY72S*	*FT*-like	1	1	3	8	2	0	14
*Araip344D4*	*TFL1*-like	2	1	4	4	2	0	13
*AraipWF9GZ*	*MFT*-like	4	3	5	8	2	0	19
*AraipPC28F*	*FT*-like	1	1	4	6	2	0	12
*AraipU9HL1*	*FT*-like	0	1	5	6	2	2	19
*AraipT1SIZ*	*FT*-like	1	3	3	6	2	1	13
*AraipYA5YU*	*FT*-like	0	3	4	9	2	1	16
*AraipA5PDN*	*TFL1*-like	1	1	3	6	2	2	17
*Araip4V81G*	*TFL1*-like	3	1	4	6	2	0	16
*AraipZJ9GZ*	*FT*-like	0	3	2	8	2	2	21
*AraipV23ZE*	*FT*-like	2	2	2	8	2	0	10
*Araip03WUR*	*FT*-like	3	2	4	5	2	1	14
*AraipT6XJY*	*TFL1*-like	2	3	5	9	3	0	18
*AraipWWI38*	*FT*-like	0	1	3	9	2	0	13
*AraipA8S33*	*MFT*-like	1	4	7	9	2	1	17
*AraipV0B0S*	*MFT*-like	0	3	5	8	2	0	16
*AraipVEP8T*	*FT*-like	1	2	4	7	2	0	14

### Wild peanut *PEBP* gene expression patterns in multiple tissues

**Fig. 6:**
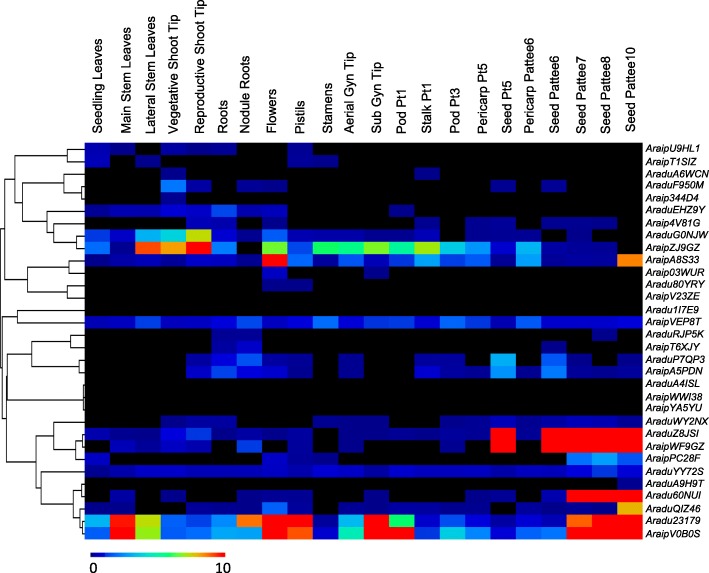
Expression profiles of 32 *PEBP* genes from two wild peanut species in 22 different tissues. Twenty-two peanut tissues (as described by Clevenger [51]) were used. For heat map analysis, *A. hypogaea* gene expression was mapped to *A. duranensis* and *A. ipaensis*, respectively

To further investigate the potential functions of wild peanut *PEBP* genes, *PEBP* gene transcription patterns were analyzed in 22 different tissues using the datasets of the *A. hypogaea* gene expression mapped to *A. duranensis* and *A. ipaensis* [51, 52]. The expression levels of several randomly selected *PEBP* genes were checked in several wild peanut and cultivated peanut tissues and found that their expression patterns were similar to the published datasets (Additional file 7). Many wild peanut *PEBP* genes showed tissue specific expression profiles. For example, all of the *MFT*-like members, including 4 *AdPEBP* members (*Aradu60NUI*, *AraduQIZ46*, *Aradu23179*, and *AraduZ8JSI*), and 3 *AiPEBP* genes (*AraipV0B0S*, *AraipWF9GZ* and *AraipA8S33*), were highly abundant in seeds, suggesting they may function in seed growth and development (Fig. 6). The *FT*-like gene *AraipZJ9GZ* showed high expression levels in lateral stem leaves, vegetative shoot tips, and reproductive shoot tips. In contrast, 5 *FT*-like members, including 2 *AdPEBP* genes (*Aradu1I7E9* and *AraduA4ISL*) and 3 *AiPEBP* genes (*AraipWWI38, AraipYA5YU,* and *AraipV23ZE*), were expressed at low levels in all tested tissues (Fig. 6), suggesting that they might have weak or no function in flowering time regulation. Duplicated gene pair expressions were also investigated and some duplicated gene pairs showed similar expression patterns in some tissues (Fig. 6). For example, *Aradu80YRY* and *AraduEHZ9Y* showed similar expression levels in flowers, and *AraduWY2NX* and *AraduF950M* closely resembled each other in the reproductive shoot tip, suggesting they may have similar functions in these tissues. In contrast, some duplicated gene pairs showed distinct expression patterns. For example, *AraduA6WCN* was expressed at high levels in the vegetative shoot tip and stalk pt1, while its duplicated gene, *AraduA4ISL,* had low expression levels in all of the tested tissues (Fig. 6), suggesting functional divergence during evolution. Because many wild peanut *PEBP* genes were orthologous with cultivated peanut *PEBP* genes (Additional file 8), orthologous gene expression levels were compared*.* Many orthologous genes had similar expression levels. For example, *AraipYA5YU/ArahyFW8Z6T*, *AraipWWI38/Arahy5H2LSK,* and *Aradu1I7E9/ArahyXGVA1E* had similar expression patterns in most of the tested tissues (Additional file 9).

### Expression analysis of wild peanut *PEBPs* under long day and short day conditions during different growth phases

**Fig. 7:**
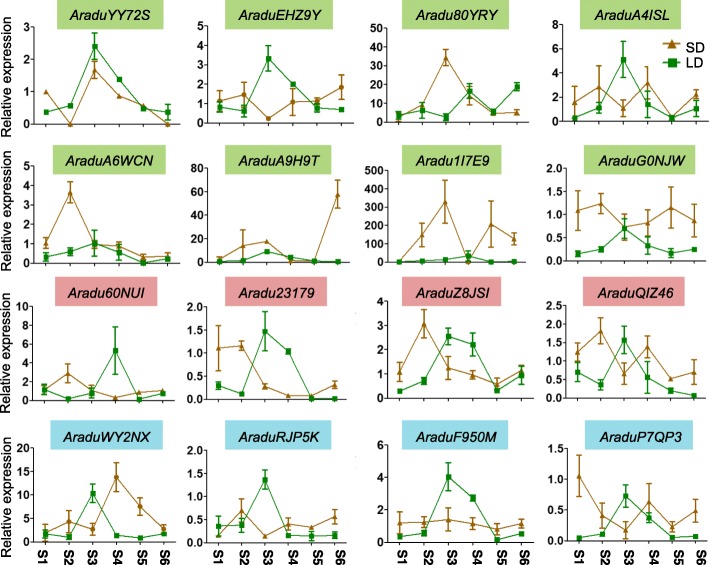
*A. duranensis* leaf *PEBP* gene expression levels under long day and short day photoperiods. LD, long day; SD, short day. The growth conditions were set as 16 h light/8 h dark and 10 h light/14 h dark cycles for the long day and short day photoperiods, respectively. The expression levels of each gene were determined relative to an *actin*-expressing gene. Each sample was analyzed using three biological replicates

**Fig. 8:**
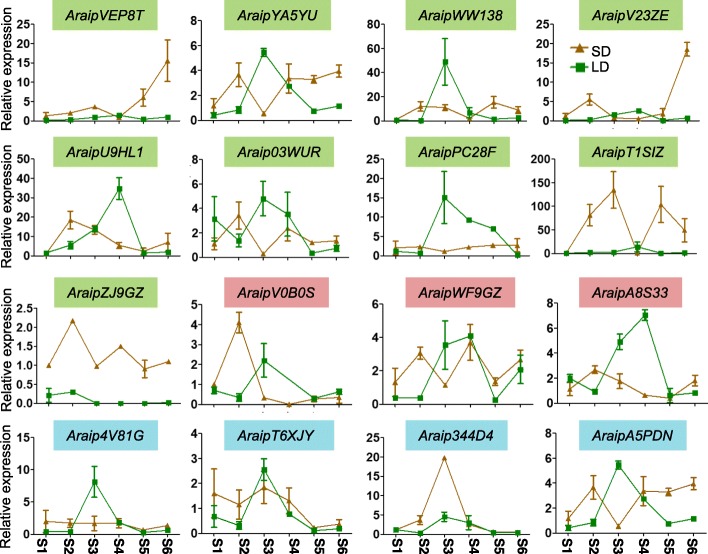
*A. ipaensis* leaf *PEBP* gene expression levels under long day and short day photoperiods. LD, long day; SD, short day. The growth conditions were set as 16 h light/8 h dark and 10 h light/14 h dark cycles for the long day and short day photoperiods, respectively. The expression levels of each gene were determined relative to an *actin*-expressing gene. Each sample was analyzed using three biological replicates

Many light-responsive *cis*-acting elements were found in wild peanut *PEBP* promoter regions. Thus, the *PEBP* gene expressions were investigated in wild peanuts under different photoperiods during different growth phases. At some growth stages, different photoperiods changed the expression patterns of most *PEBP* genes (Figs. 7 and 8), suggesting they displayed important roles in light responsiveness during related stages of development. Some *PEBP* genes showed consistent expression levels during all growth phases under long day conditions, including *AraduA6WCN*, *AraduA9H9T,* and *Aradu1I7E9* in *A. duranensis*, and *AraipVEP8T*, *AraipV23ZE*, *AraipT1SIZ,* and *AraipZJ9GZ* in *A. ipaensis*, all of which belong to the *FT*-like sub-family (Figs. 2, 7 and 8). Under the short day photoperiod, *AraduF950M*, *AraipPC28F,* and *Araip4V81G* showed consistent expression levels in different growth phases (Figs. 7 and 8). In addition, the expression of *AraipZJ9GZ* was higher under short day conditions than under long day conditions in all tested growth stages (Fig. 8). Some duplicated gene pairs displayed similar expression patterns throughout the growth phases under long day conditions, such as *AraduYY72S/AraduEHZ9Y* and *AraipWWI38/AraipYA5YU* (Figs. 7 and 8), suggesting these duplicated genes may be functionally redundant.

## Discussion

Characterization of *PEBP* genes has greatly increased our knowledge of the molecular mechanisms regulating flowering time and plant architecture in many plant species [8, 18–20]. Peanut is an important oilseed crop worldwide, and the identification of peanut *PEBP* genes helps to further our functional understanding of peanut flowering time and plant architecture regulation. Additionally, the investigation of wild peanut *PEBP* genes provides essential information for further functional characterization of related genes in cultivated peanuts [40, 41]. In our study, thirty-two *PEBP* genes were identified and characterized from two wild peanut genomes.

Different plant species have different numbers of *PEBP* genes [8, 18, 19]. Each of the two diploid wild peanut species, *A. duranensis* and *A. ipaensis*, has 16 *PEBP* genes (Table 1). The allotetraploid cultivated peanut is thought to be derived from hybridization and polyploidization of the two diploid wild species, and the genome size of *A. hypogaea* is close to the sum of *A. duranensis* and *A. ipaensis* genomes [36–39]. The *A. hypogaea* genome contains 31 *PEBP* members (Additional file 2), which is also close to the sum of the *PEBP* genes in the *A. duranensis* and *A. ipaensis* genomes. The two wild peanut genomes contain 8 *TFL1*-like, 17 *FT*-like, and 7 *MFT*-like members (Fig. 2 and Table 1), while the *A. hypogaea* genome contains 8 *TFL1*-like, 16 *FT*-like, and 7 *MFT*-like members (Additional file 2). Likely, one of the *FT*-like genes in *A. hypogaea* was lost during evolution. In addition, Arabidopsis, rice, soybean, and maize have 6, 19, 23, and 24 *PEBP* members, respectively [8, 18, 19]. The genome sizes of *A. duranensis*, *A. ipaensis*, *A. hypogaea,* Arabidopsis, rice, soybean, and maize are 1.25 Gb, 1.56 Gb, 2.7 Gb, 125 Mb, 466 Mb, 1.1 Gb, and 2.3 Gb, respectively [40, 62–65]. Thus, the genome sizes in these plants are not correlated with the number of *PEBP* genes.

Gene duplication is particularly prevalent in plants and provides resources for novel gene functions. Some duplicated genes become pseudogenized and have no function, while other gene duplication pairs evolve new functions [8, 66]. For example, duplicated *PEBP* genes in soybean have functionally diverged due to the alteration of critical amino acids [8]. Four interchromosomal duplications and one tandem duplication were identified in *A. duranensis*, and two interchromosomal duplications and one tandem duplication were found in *A. ipaensis* (Fig. 5). Some of these wild peanut duplicated gene pairs showed similar expression patterns, while the expression levels of others differed across tissues or growth phases (Figs. 6, 7 and 8). For example, *Aradu80YRY*/*AraduEHZ9Y* and *AraduWY2NX*/*AraduF950M* showed similar expression levels in the reproductive shoot tip, suggesting the functional conservation of these genes. In contrast, expression of *AraduA6WCN* was high in the vegetative shoot tip and stalk pt1, while its duplicate, *AraduA4ISL,* had low expression levels in those two tissues (Fig. 6), suggesting functional divergence of these duplicated *PEBP* genes.

Among these *PEBP* members, *FT* and *TFL1* are well studied in many species [1]. The regulation of flowering time by *FT* has been highly investigated. The *FT*-like genes *Aradu80YRY, AraduYY72S,* and *AraduEHZ9Y* in *A. duranensis*, and *AraipV23ZE, AraipVEP8T, AraipYA5YU,* and *AraipWWI38* in *A. ipaensis*, are highly conserved with Arabidopsis *FT* and *TSF* genes (Fig. 2). However, *AraipV23ZE*, *AraipYA5YU,* and *AraipWWI38* had very low expression levels in all tested tissues (Fig. 6). It is likely that *Aradu80YRY, AraduYY72S,* and *AraduEHZ9Y* in *A. duranensis* and *AraipVEP8T* in *A. ipaensis* have important roles in flowering time regulation. *TFL1* is one of the most important genes involved in plant architecture regulation. Genes homologous to *TFL1* have been shown to be involved in plant architecture regulation in many legumes, such as soybean (*GmDt1*), common bean (*PvTFL1y*), and mungbean (*VrDet1*), and these three genes are syntelogs [1, 58–61]. Synteny between *AraduF950M*/*AraduWY2NX*, *Araip344D4*/*Araip4V81G, GmDt1*, and *PvTFL1y* were observed in *A. duranensis* and *A. ipaensis*, respectively (Additional file 5), suggesting that they function in plant architecture regulation. There is only one *TFL1* gene involved in plant architecture regulation in soybean, common bean, and mungbean. Although two genes have synteny with soybean *GmDt1* and common bean *PvTFL1y* in *A. duranensis* and *A. ipaensis*, respectively, it is possible that there is only one key member involved in plant architecture regulation in either of the two wild peanut species. *MFT* has an important role in seed germination via the abscisic acid and gibberellic acid pathways [34, 35]. In wild peanuts, *MFT-*like *PEBP* members were expressed at higher levels in seeds than in other tissues (Fig. 6), suggesting that these genes might play critical roles in seed development and seed germination pathways. Future work is needed to fully elucidate the involvement of *PEBP* genes in flowering time and plant architecture regulation pathways in wild peanuts.

*Cis*-acting elements are important factors that bind transcription factors and active gene expression. Peanut *PEBP* promoter regions were found to contain a variety of *cis-*acting elements, including developmental elements, environmental stress related elements, hormone-responsive elements, light-responsive elements, promoter related elements, and site-binding related elements (Table 3 and Additional file 6). This suggests that *PEBP* genes might have critical roles in these signal pathways. Additionally, expression patterns of *PEBP* genes varied in different tissues and different growth phases (Figs. 6, 7 and 8), further supporting functional differences. All of the *PEBP* genes contained light-responsive elements, and most of the peanut *PEBP* genes showed altered expression patterns under long day versus short day growth conditions (Figs. 7, 8 and Table 3), indicating that they might be involved in plant development via photoperiod dependent pathways. Although most of the *FT*-like genes contained these 6 types of functional *cis*-acting elements (Table 3 and Additional file 6), 10 *FT*-like members had either low expression levels in different tissues or unchanged expression levels across growth stages under long day conditions (Figs. 6, 7 and 8). This could be due to the fact that these genes have weak or no function in these tissues or growth stages under long day photoperiod conditions. Some *PEBP* members, such as *FT*-like genes [1, 23, 49], are circadian clock genes and might be expressed at other times of day that were not tested in this study. Moreover, the *FT*-like gene *AraipZJ9GZ* showed higher expression levels under short day conditions (Fig. 8), suggesting that *AraipZJ9GZ* is involved in flowering time regulation under short day rather than long day conditions. In all, the variation in *cis*-element numbers and gene expression patterns of *FT*-like genes likely reflects their functional diversities in flowering time regulation. *TFL1*-like and *MFT*-like *PEBP* genes are likely similarly diverse in other functional pathways. Additionally, many wild peanut *PEBP* genes were found to be orthologous to cultivated peanut genes (Additional file 8), thus, the expression patterns of wild peanut *PEBP* genes can be used to deduce *PEBP* gene expression in *A. hypogaea* during growth phases under long day and short day conditions.

## Conclusions

Genome-wide analysis was used to identify and characterize 16 *PEBP* genes from each of the two diploid wild peanut species, *A. duranensis* and *A. ipaensis*. Many characteristics of these *PEBP* genes were investigated, including chromosomal distributions, gene structures, and motifs. The 32 *PEBP* genes were classified into *TFL1*-like, *FT*-like, and *MFT*-like sub-families. Interchromosomal duplicated gene pairs and tandem duplication events were identified in both wild peanut species. In addition, four genes that are likely to play important roles in plant architecture and another four that likely regulate flowering time were identified. Ninety-five *cis*-acting elements were identified, 56 of which have putative functions and may be responsible for tissue and photoperiod expression pattern differences. Detailed understanding of wild peanut *PEBP* genes will be useful for future efforts to modify peanut plant flowering time and plant architecture.

## Supplementary information

**Additional file 1.** Subcellular localizations of PEBP proteins in tobacco leaf cells.

**Additional file 2.** Evolutionary relationship analysis of PEBP proteins from wild and cultivated peanuts.

**Additional file 3. **Alignment of wild peanut PEBP domains. The wathet, chrysoidine, and aqua colors indicate *TFL1*-like, *MFT*-like and *FT*-like sub-families, respectively.

**Additional file 4.** Sequence logos of 15 motifs in wild peanut PEBP proteins. The “sites” and “width” indicate the number of wild peanut PEBP proteins containing each motif and the amino acid number of each motif, respectively.

**Additional file 5. **Synteny analysis between soybean *GmDt1*, common bean *PvTFL1y,* and wild peanut *TFL1*-like genes. Syntenic regions surrounding the analyzed homologous genes between soybean, common bean, and wild peanuts were investigated. (a) Synteny analysis between *AraduRJP5K*, *AraipT6XJY,* and *GmDt1 (Glyma19g194300)*, and *PvTFL1y (Phvul001g189200).* (b) Synteny analysis between *AraduF950M*, *Araip344D4, GmDt1*, and *PvTFL1y.* (c) Synteny analysis between *AraduWY2NX*, *Araip4V81G, GmDt1*, and *PvTFL1y.* The red boxes indicate our target genes and the green boxes indicate genes surrounding the homologous genes. Gm, *Glycine max*; Pv, *Phaseolus vulgaris.*

**Additional file 6. **Functions of the *cis*-acting elements found in the promoter regions of wild peanut *PEBP* genes.

**Additional file 7. **Expression patterns of several wild and cultivated peanut *PEBP* genes.

**Additional file 8.** Wild and cultivated peanut orthologous genes.

**Additional file 9. **Expression profiles of *PEBP* genes in 22 different tissues from cultivated and wild peanut species.

**Additional file 10.** Primers used in this study.

## Data Availability

The datasets generated and analyzed during the current study are available from the corresponding author upon reasonable request.

## Abbreviations

A. duranensis: Arachis duranensis
A. ipaensis: Arachis ipaensis
AA: Amino acid
ATC: *Arabidopsis thaliana* CENTRORADIALIS
BFT: BROTHER OF FT
CDS: Coding domain sequence
Chr: Chromosome number
FT: FLOWERING LOCUS T
Gm: *Glycine max*
GSDS: Gene Structure Display Server program
LD: Long day; SD, short day
MFT: MOTHER OF FT AND TFL1
Mol.Wt: Molecular weight
N/A: Not applicable
NCBI: National Center for Biotechnology Information
PEBP: Phosphatidyl ethanolamine-binding protein
pI: Isoelectric point
Pv: *Phaseolus vulgaris*
qRT-PCR: Quantitative real-time PCR
S1: Stage 1
S2: Stage 2
S3: Stage 3
S4: Stage 4
S5: Stage 5
S6: Stage 6
TFL1: TERMINAL FLOWER1
TSF: TWIN SISTER OF FT
