# Supplementary figures and images for: Molecular and transcriptional characterization of phosphatidyl ethanolamine-binding proteins in wild peanuts Arachis duranensis and Arachis ipaensis
Source: BMC Plant Biol. 2019 Nov 9;19:484. doi: 10.1186/s12870-019-2113-3 (PMC6842551; doi:10.1186/s12870-019-2113-3)

## Slide 1
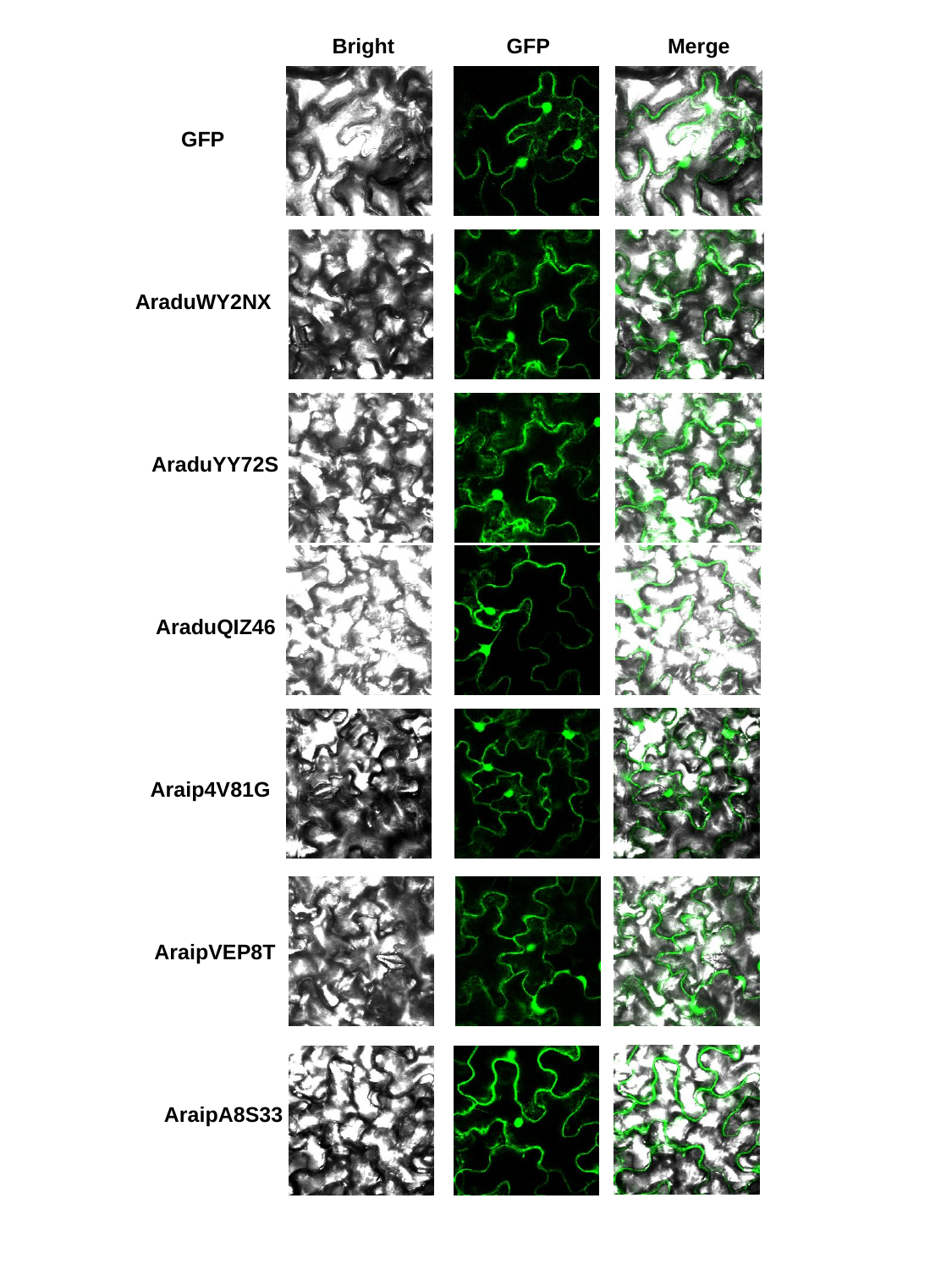

Bright GFP Merge
GFP
AraduWY2NX
AraduYY72S
AraduQIZ46
Araip4V81G
AraipVEP8T
AraipA8S33

Supplement: Supplementary file 1 — Additional file 1. Subcellular localizations of PEBP proteins in tobacco leaf cells. [file 12870_2019_2113_MOESM1_ESM.pptx]

## Slide 1
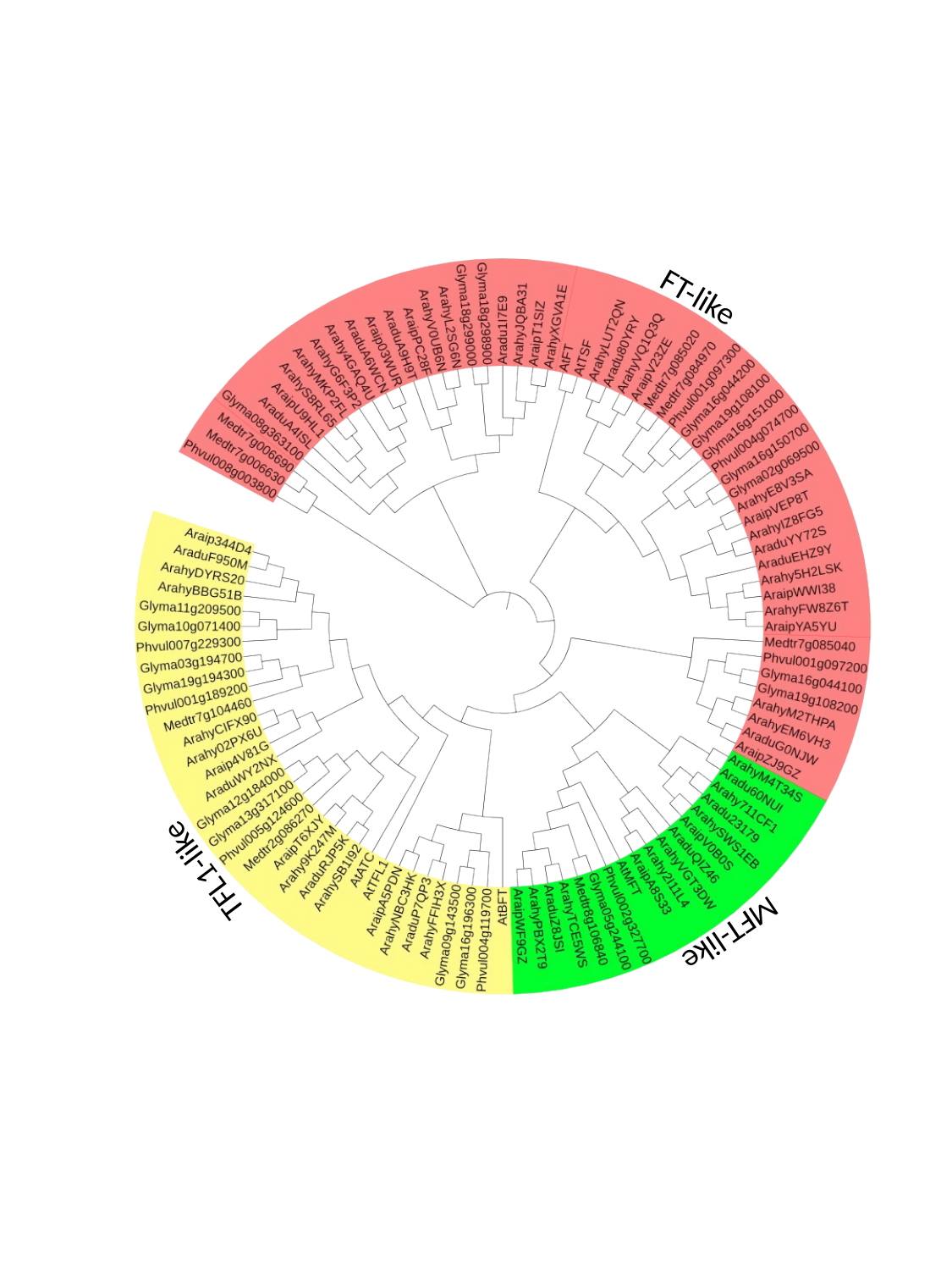

FT-like
TFL1-like
MFT-like

Supplement: Supplementary file 2 — Additional file 2. Evolutionary relationship analysis of PEBP proteins from wild and cultivated peanuts. [file 12870_2019_2113_MOESM2_ESM.pptx]

## Slide 1
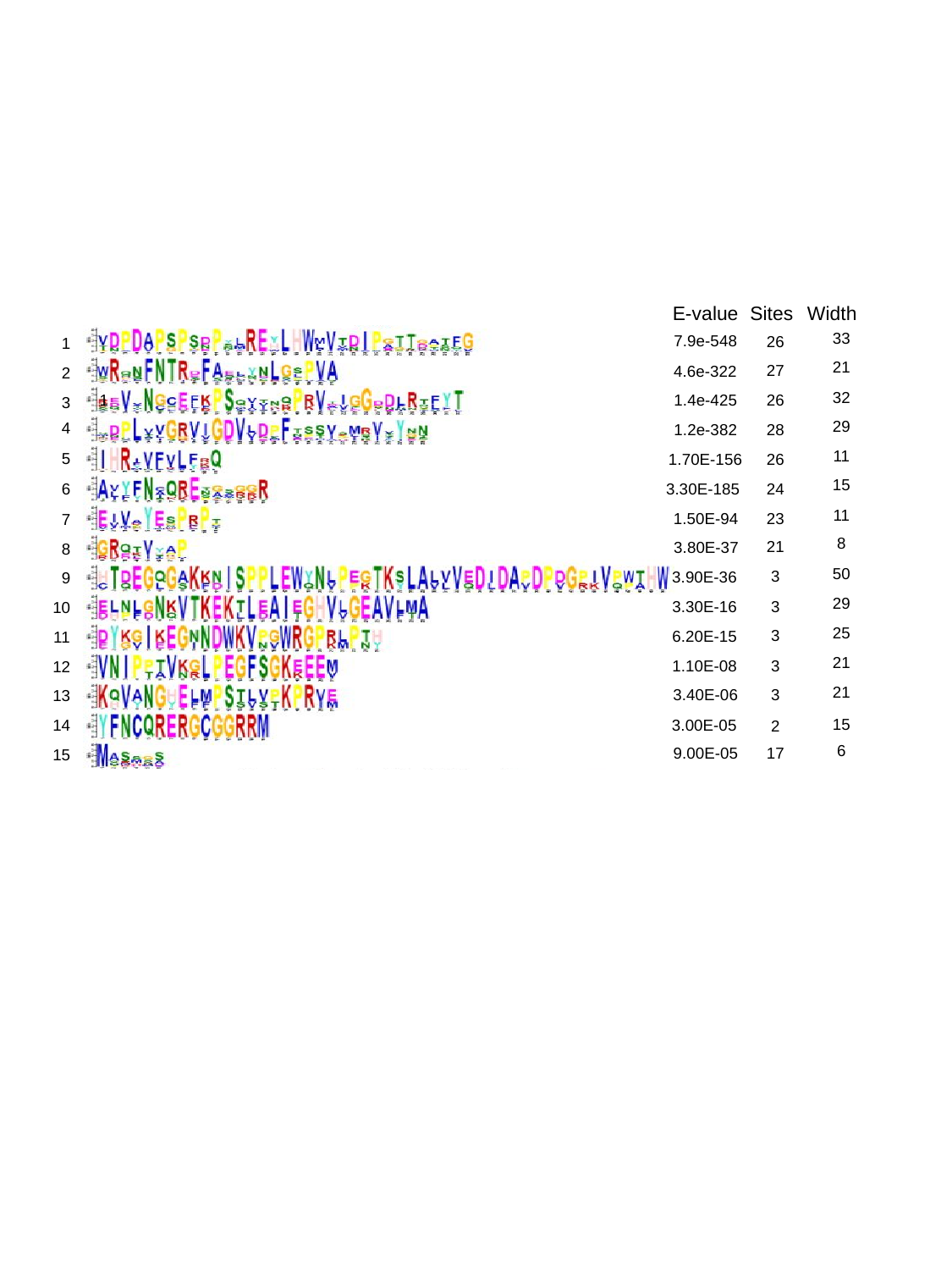

E-value
Sites
Width
33
7.9e-548
26
1
21
27
4.6e-322
2
32
26
1.4e-425
1
3
29
4
28
1.2e-382
11
5
26
1.70E-156
15
3.30E-185
6
24
11
1.50E-94
23
7
8
21
3.80E-37
8
50
3
3.90E-36
9
29
3.30E-16
3
10
25
3
6.20E-15
11
21
1.10E-08
3
12
21
3.40E-06
3
13
15
3.00E-05
14
2
6
17
9.00E-05
15

Supplement: Supplementary file 4 — Additional file 4. Sequence logos of 15 motifs in wild peanut PEBP proteins. The “sites” and “width” indicate the number of wild peanut PEBP proteins containing each motif and the amino acid number of each motif, respectively. [file 12870_2019_2113_MOESM4_ESM.pptx]

## Slide 1
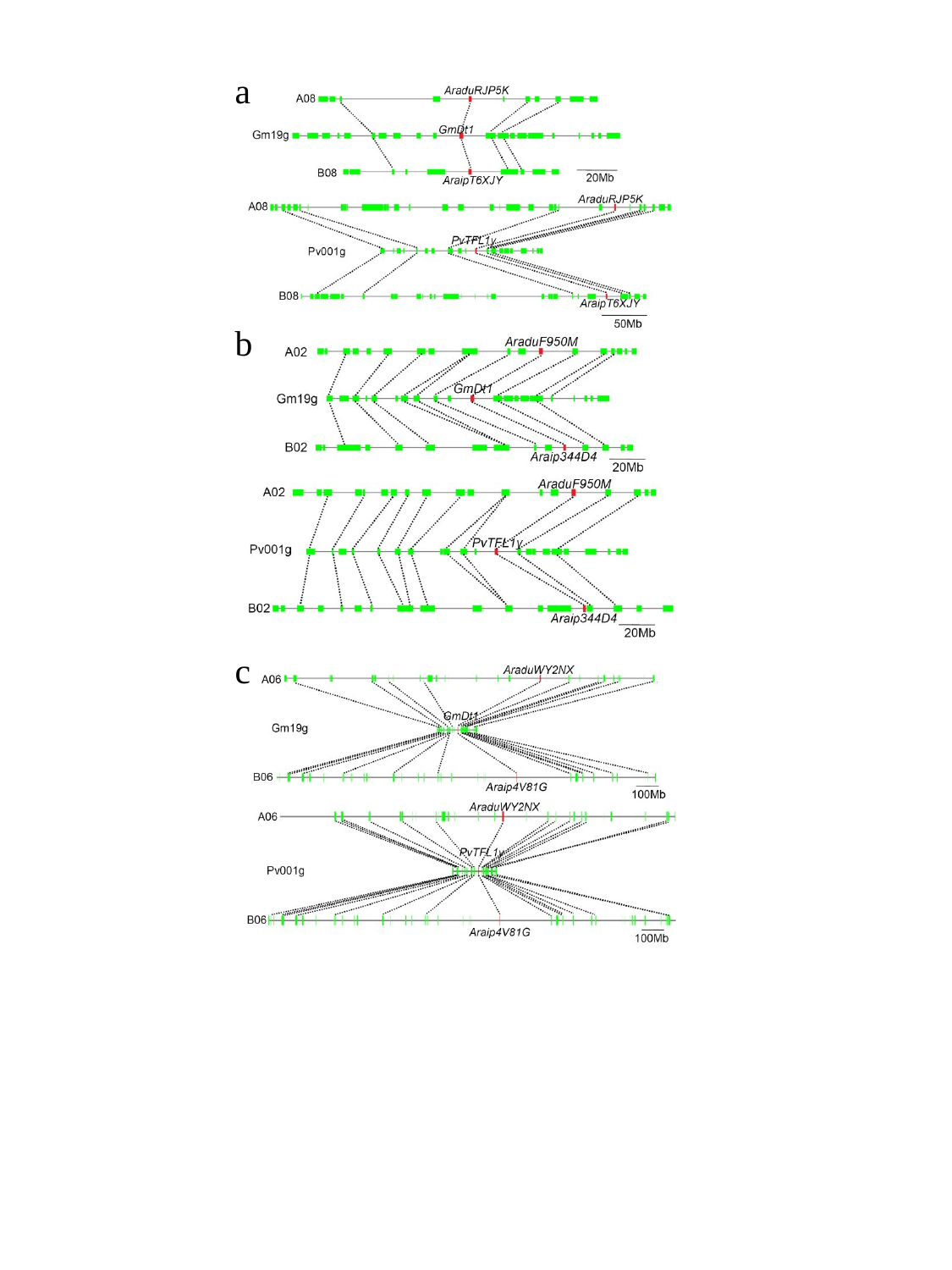

a
b
c

Supplement: Supplementary file 5 — Additional file 5. Synteny analysis between soybean GmDt1, common bean PvTFL1y, and wild peanut TFL1-like genes. Syntenic regions surrounding the analyzed homologous genes between soybean, common bean, and wild peanuts were investigated. (a) Synteny analysis between AraduRJP5K, AraipT6XJY, and GmDt1 (Glyma19g194300), and PvTFL1y (Phvul001g189200). (b) Synteny analysis between AraduF950M, Araip344D4, GmDt1, and PvTFL1y. (c) Synteny analysis between AraduWY2NX, Araip4V81G, GmDt1, and PvTFL1y. The red boxes indicate our target genes and the green boxes indicate genes surrounding the homologous genes. Gm, Glycine max; Pv, Phaseolus vulgaris. [file 12870_2019_2113_MOESM5_ESM.pptx]
