# Supplementary material for: Molecular and transcriptional characterization of phosphatidyl ethanolamine-binding proteins in wild peanuts Arachis duranensis and Arachis ipaensis
Source: BMC Plant Biol. 2019 Nov 9;19:484. doi: 10.1186/s12870-019-2113-3 (PMC6842551; doi:10.1186/s12870-019-2113-3)
Supplement: Supplementary file 3 — Additional file 3. Alignment of wild peanut PEBP domains. The wathet, chrysoidine, and aqua colors indicate TFL1-like, MFT-like and FT-like sub-families, respectively. [file 12870_2019_2113_MOESM3_ESM.pptx]

## Slide 1
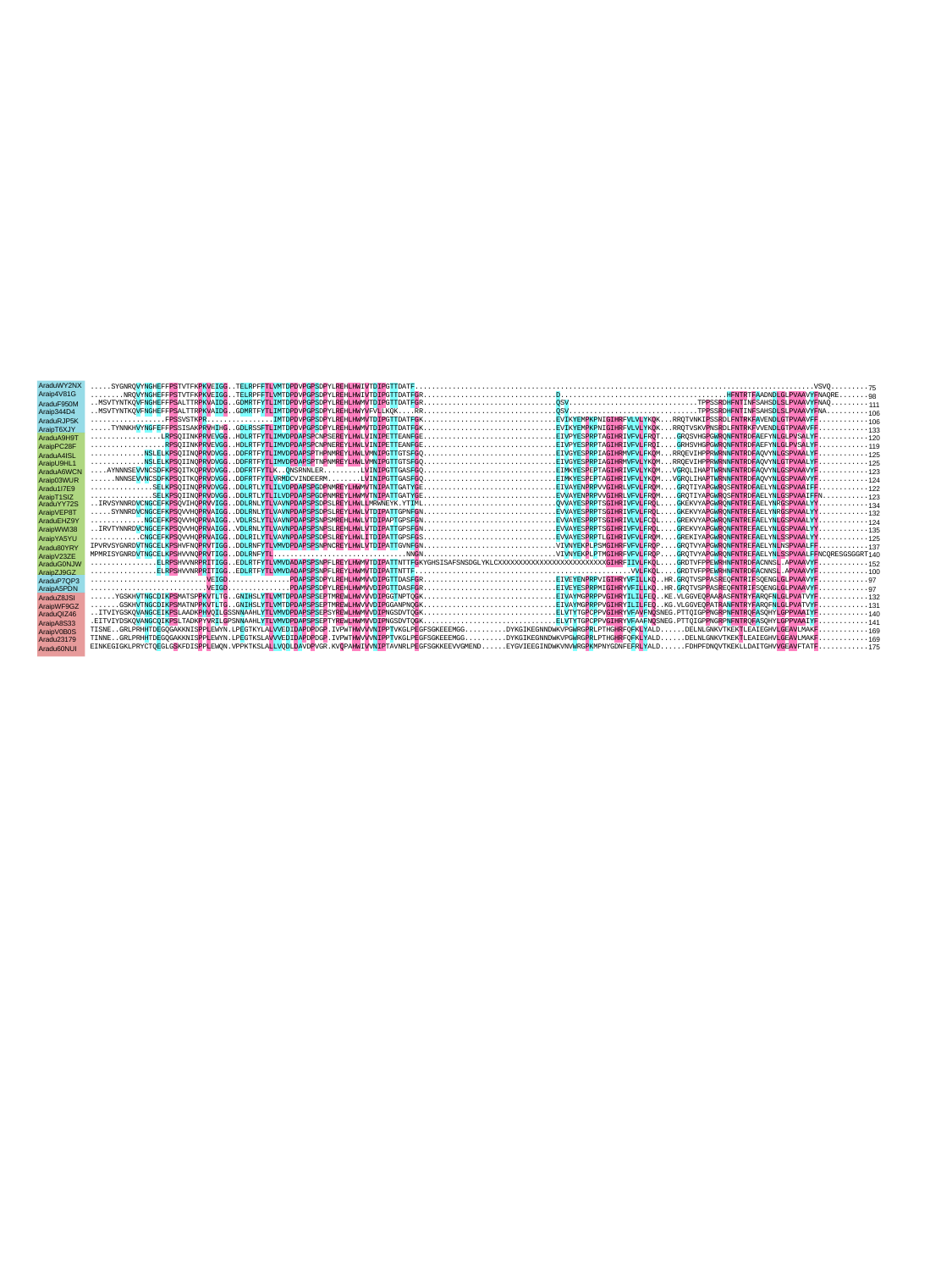

AraduWY2NX
75
Araip4V81G
98
AraduF950M
111
Araip344D4
106
AraduRJP5K
106
AraipT6XJY
133
AraduA9H9T
120
119
AraipPC28F
125
AraduA4ISL
125
AraipU9HL1
123
AraduA6WCN
124
Araip03WUR
122
Aradu1I7E9
AraipT1SIZ
123
AraduYY72S
134
AraipVEP8T
132
AraduEHZ9Y
124
AraipWWI38
135
125
AraipYA5YU
137
Aradu80YRY
140
AraipV23ZE
AraduG0NJW
152
100
AraipZJ9GZ
97
AraduP7QP3
AraipA5PDN
97
132
AraduZ8JSI
131
AraipWF9GZ
140
AraduQIZ46
141
AraipA8S33
169
AraipV0B0S
169
Aradu23179
175
Aradu60NUI
