# Supplementary material for: Molecular and transcriptional characterization of phosphatidyl ethanolamine-binding proteins in wild peanuts Arachis duranensis and Arachis ipaensis
Source: BMC Plant Biol. 2019 Nov 9;19:484. doi: 10.1186/s12870-019-2113-3 (PMC6842551; doi:10.1186/s12870-019-2113-3)
Supplement: Supplementary file 7 — Additional file 7. Expression patterns of several wild and cultivated peanut PEBP genes. [file 12870_2019_2113_MOESM7_ESM.pptx]

## Slide 1
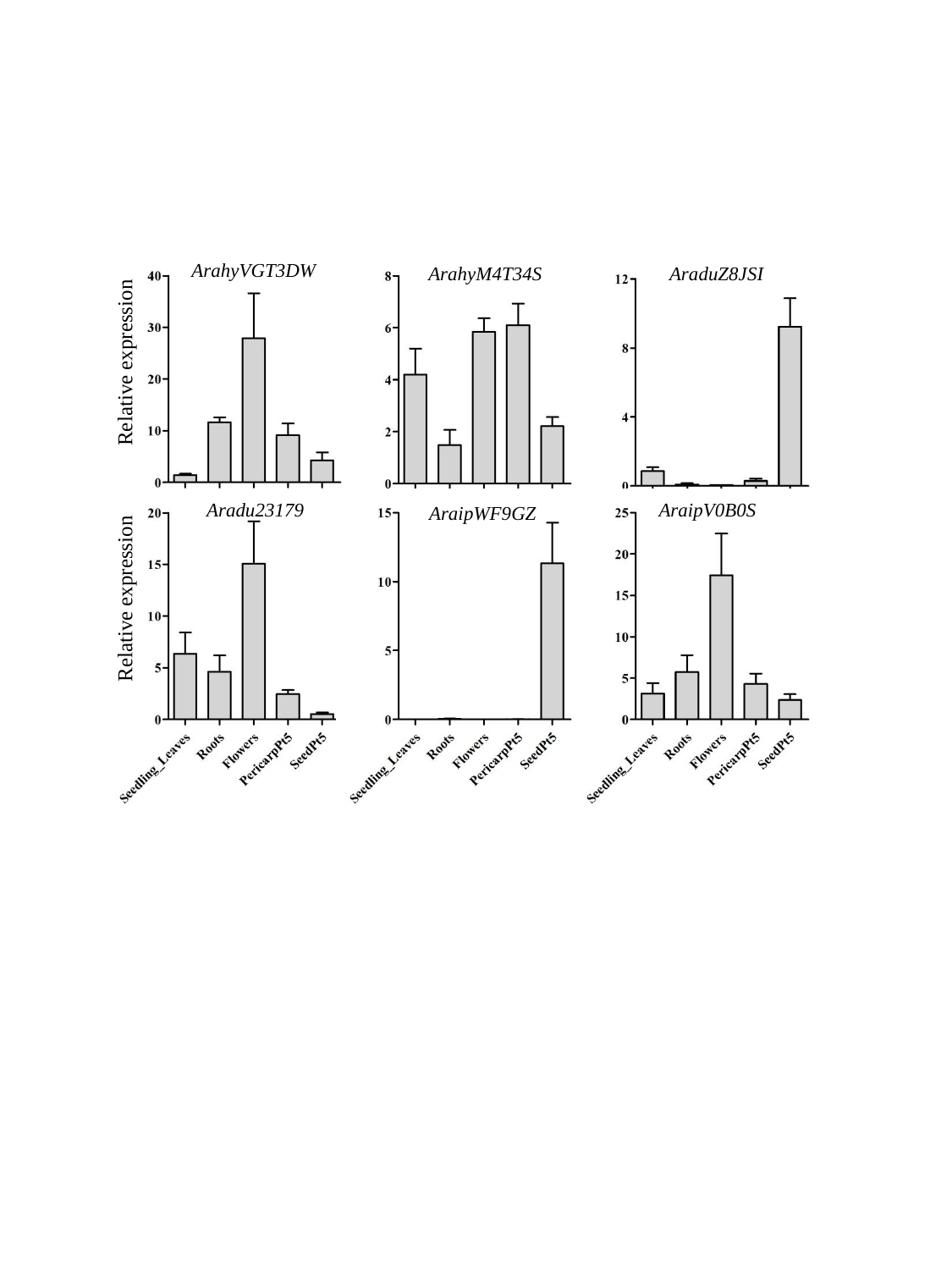

Relative expression
ArahyVGT3DW
ArahyM4T34S
AraduZ8JSI
Relative expression
Aradu23179
AraipV0B0S
AraipWF9GZ
