# Supplementary material for: Molecular and transcriptional characterization of phosphatidyl ethanolamine-binding proteins in wild peanuts Arachis duranensis and Arachis ipaensis
Source: BMC Plant Biol. 2019 Nov 9;19:484. doi: 10.1186/s12870-019-2113-3 (PMC6842551; doi:10.1186/s12870-019-2113-3)
Supplement: Supplementary file 9 — Additional file 9. Expression profiles of PEBP genes in 22 different tissues from cultivated and wild peanut species. [file 12870_2019_2113_MOESM9_ESM.pptx]

## Slide 1
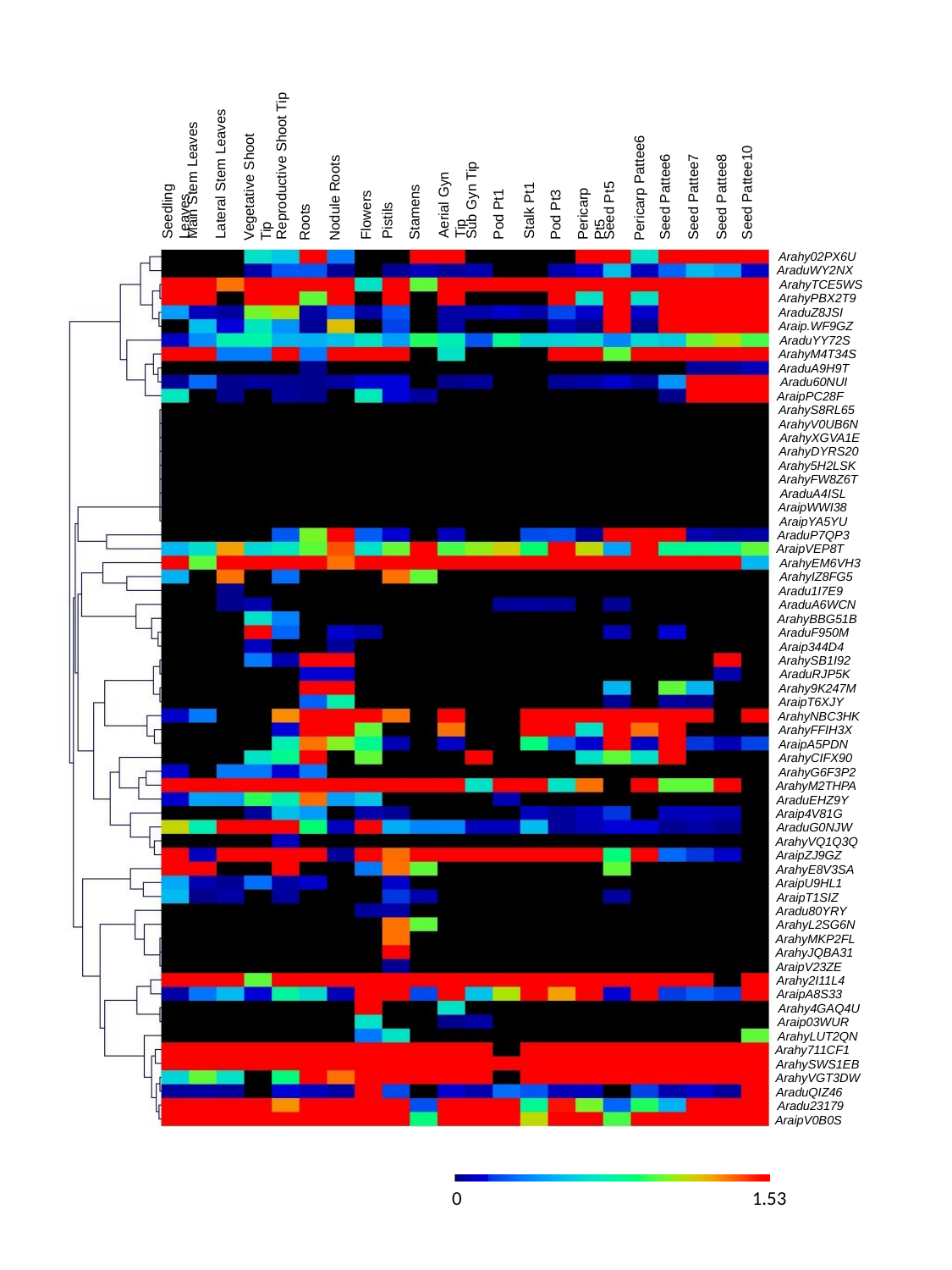

Roots
Reproductive Shoot Tip
Lateral Stem Leaves
Vegetative Shoot Tip
Main Stem Leaves
Pericarp Pattee6
Flowers
Nodule Roots
Seedling Leaves
Seed Pattee7
Seed Pattee10
Seed Pattee6
Sub Gyn Tip
Seed Pattee8
Aerial Gyn Tip
Pod Pt1
Pod Pt3
Pericarp Pt5
Stamens
Seed Pt5
Pistils
Stalk Pt1
Arahy02PX6U
AraduWY2NX
ArahyTCE5WS
ArahyPBX2T9
AraduZ8JSI
Araip.WF9GZ
AraduYY72S
ArahyM4T34S
AraduA9H9T
Aradu60NUI
AraipPC28F
ArahyS8RL65
ArahyV0UB6N
ArahyXGVA1E
ArahyDYRS20
Arahy5H2LSK
ArahyFW8Z6T
AraduA4ISL
AraipWWI38
AraipYA5YU
AraduP7QP3
AraipVEP8T
ArahyEM6VH3
ArahyIZ8FG5
Aradu1I7E9
AraduA6WCN
ArahyBBG51B
AraduF950M
Araip344D4
ArahySB1I92
AraduRJP5K
Arahy9K247M
AraipT6XJY
ArahyNBC3HK
ArahyFFIH3X
AraipA5PDN
ArahyCIFX90
ArahyG6F3P2
ArahyM2THPA
AraduEHZ9Y
Araip4V81G
AraduG0NJW
ArahyVQ1Q3Q
AraipZJ9GZ
ArahyE8V3SA
AraipU9HL1
AraipT1SIZ
Aradu80YRY
ArahyL2SG6N
ArahyMKP2FL
ArahyJQBA31
AraipV23ZE
Arahy2I11L4
AraipA8S33
Arahy4GAQ4U
Araip03WUR
ArahyLUT2QN
Arahy711CF1
ArahySWS1EB
ArahyVGT3DW
AraduQIZ46
Aradu23179
AraipV0B0S
0
1.53
